# Supplementary material for: Plasma IL-1 and IL-6 Family Cytokines with Soluble Receptor Levels at Diagnosis in Head and Neck Squamous Cell Carcinoma: High Levels Predict Decreased Five-Year Disease-Specific and Overall Survival
Source: Cancers (Basel). 2024 Apr 12;16(8):1484. doi: 10.3390/cancers16081484 (PMC11048558; doi:10.3390/cancers16081484)
Supplement: Supplementary file 1 [file cancers-16-01484-s001.zip › cancers-2951350-supplementary.pdf]

Variable		Distribution
Age at diagnosis	Years (Mean ± SD)	57.7 ± 9.5
Gender	Males	46
	Females	11
T stage	0	4
	1	13
	2	29
	3	5
	4	6
N stage	0	11
	1	13
	2	31
	3	2
Smoking	Never smoked	24
	< 10 pack years	7
	Probably < 10 pack years	6
	Probably > 10 pack years	4
	> 10 pack years	16
	Missing	0
Site (ICD-10)	Oropharynx	54
	Oral Cavity	1
	Other	2
Total patients		57

Supplementary Table S1. Clinical HPV(+) patient characteristics at diagnosis.

Analyte	Minimum	25th Perc. (pg/mL)	Median (pg/mL)	75th Perc. (pg/mL)	Maximum
IL-6	.20	1.25	1.87	2.80	15.9
s-gp130	40175	67944	82014	88928	108956
s-IL6-Rα	5119	27041	32860	37164	47290
IL1-RA	119	306	381	581	3185
IL-31	19.4	57.56	71.24	85.41	132
IL-33Rα/ST2	76	14242	17520	22920	107876

Supplementary Table S2: Concentration and Quartile Values of Measured Cytokines and Soluble Cytokine Receptors. The table displays the actual concentration of quartile values (25th percentile, median and 75th percentile) as well as minimum and maximum values determined for the measured cytokines and soluble cytokine receptors in our assay. All values are reported in picograms per milliliter (pg/mL).
